# ‘Do You Tell Them Not to Kiss Their Child?’ Implementing Congenital Cytomegalovirus Prevention Guidelines in General Practice: A Qualitative Study

**DOI:** 10.1111/ajo.70141

**Published:** 2026-04-28

**Authors:** Natalia Rode, Hayley Smithers‐Sheedy, Kath Swinburn, Tanya Tripathi, Emma Waight, Lisa Hui

**Affiliations:** ^1^ Department of Obstetrics, Gynaecology and Newborn Health The University of Melbourne Melbourne Victoria Australia; ^2^ Cerebral Palsy Alliance Research Institute The University of Sydney Sydney New South Wales Australia

**Keywords:** cytomegalovirus, general practitioner, practice guidelines, pregnancy, qualitative research

## Abstract

Congenital cytomegalovirus (CMV) is a known cause of childhood‐onset disability. Australian guidelines recommend all pregnant women be informed about CMV risk‐reduction strategies. We conducted semi‐structured interviews with six Australian General Practitioners (GPs) who had completed a CMV eLearning module to explore their education experience and identify barriers and facilitators to including CMV in routine antenatal counselling. Data were analysed inductively. GPs found the module ‘engaging’ and ‘relevant’ to their practice. Barriers to routine CMV antenatal counselling included *system*, *practitioner* and *patient* factors, with GPs identifying measures to address these issues and facilitate routine counselling in line with national recommendations.

## 1 Introduction

Congenital cytomegalovirus (CMV) is the most common infectious cause of childhood disability in Australia, including sensorineural hearing loss, epilepsy and cerebral palsy [1, 2, 3]. Simple behavioural hygiene measures can reduce the risk of maternal CMV infection [4], and national guidelines recommend that all women who are pregnant or planning pregnancy are counselled regarding these [5, 6]. However, few maternity care providers consistently counsel women on CMV prevention [7] and awareness amongst women who are pregnant or planning pregnancy remains low [8]. General practitioners (GPs), who are often the first point of contact for preconception and antenatal care, report common barriers to counselling such as insufficient knowledge about CMV, limited consultation time, and concern about causing anxiety [7]. To address these challenges, an eLearning module on CMV and syphilis was developed for GPs [9]. This study aimed to evaluate GP experiences of the module and to identify barriers and facilitators to implementing congenital CMV prevention guidelines into routine practice.

## 2 Methods

GPs who completed the ‘Infections in Pregnancy’ eLearning module in 2023 were invited to participate in semi‐structured interviews. Six female GPs from New South Wales, Queensland, South Australia, and Tasmania participated. Interviews explored experiences with the eLearning module, perceptions of its applicability, and barriers and facilitators to CMV counselling. Data were analysed inductively by a team of four researchers, with coding discrepancies resolved through discussion. The Consolidated Criteria for Reporting Qualitative Research (COREQ) informed the design and analysis. Ethics approval was granted by the Mercy Health Human Research Ethics Committee (HREC no. 2022‐047).

## 3 Results

Three major themes emerged: course delivery, barriers to implementation, and facilitators to implementation.

### 3.1 Course Delivery

GPs described the module as engaging, practical, and highly relevant to general practice. Case studies and videos were particularly valued and assisted in clarifying both CMV transmission and risk‐based screening. Participants reported improved confidence and knowledge, with some describing changes in their antenatal counselling practice.

### 3.2 Barriers to Implementing Congenital CMV Prevention Guidelines

Barriers existed at multiple levels (Table 1). At the *system* level, lack of consultation time and insufficient Medicare funding for long antenatal visits were highlighted, along with the absence of CMV content in standard GP resources such as HealthPathways and the RACGP Redbook guidelines.

There's just too much information that you talk to patients about… on the first consult… I already have a 30‐minute consult and often it can go over, so to add that….

It would be great if antenatal consults were better Medicare‐funded… which would allow more time… and opportunity.

**TABLE 1:** Barriers to GP implementation of congenital CMV guideline recommendations.

Level 1 subcategory	Level 2 subcategory	Example quotes
Systems level barriers	Insufficient consultation time	‘In antenatal care there's a tremendous amount of things to cover and just to add that one little thing…’ ‘It's like of the 1 million things to educate people on…’
	Medicare funding insufficient for antenatal consultations	‘It would be great if antenatal consults were better Medicare‐funded…which would just allow or help encourage more time and opportunity to do that sort of thing’
	Lack of CMV information in standard GP antenatal resources	‘We all have those state books that we give patients…I don't think it is … in the Queensland one at least’. ‘Normally, I use a Murtagh handout on pregnancy planning, and that has a big section on Listeria and Toxo, and nothing on CMV’. ‘I don't have like a nice, neat handout that has, every single thing on it’
Practitioner level barriers	GP ambivalence about CMV prevention message	‘it just felt like it interfered with the mother–child bonding’
	Lack of consistent messaging amongst GPs in a practice	‘I know a lot of my colleagues still don't (provide CMV information to patients), and I guess whether it is just that confidence level or that comfort level I, I guess, the main issue… They're not yet confident’
	Desire for the message to come from outside the GP consultation room	‘I just wish maybe that information was given to mothers outside of the doctor's office…like a poster, or an app… social media… commercials’
Perceived patient level barriers	Information overload	‘The main limitation is really that, time and probably information overload for patients’
	Individual patient characteristics	‘and potentially cultural barriers as well, depending on the individual patient and their culture, and what that might entail in terms of their ideas about health and risks’. ‘you know, you really struggle to get them to follow up with routine antenatal care, let alone take routine antenatal advice’
	Perceived patient sensitivities	‘I've had some patients be funny about… my recommendations of like, “Oh maybe don't kiss your kids on the lips.” And some people would be like “of course I'm going to”’

At the *practitioner* level, GPs reported personal ambivalence about the CMV prevention message and a desire for broader public health campaigns to normalise the message.

We just don't have enough information about this topic… there are so many uncertainties and unknowns.

How do I bring it up…do you tell people not to kiss their child?

Inconsistent counselling practices amongst doctors within a practice was also noted: ‘I think having consistent messages throughout practitioners is tricky… people come back (after an initial pregnancy visit) and it hasn't been mentioned, and then it's an extra load to bring it up later’.

At the *patient* level, information overload, limited health literacy, cultural barriers, and concerns about patient anxiety or sensitivities about the medicalisation of pregnancy were reported as challenges.

There's just so much information to give them. You talk about all this stuff and then they forget half of it.

Some GPs described selectively offering CMV information based on perceived patient anxiety or risk. ‘I just have to risk stratify them…I try to do it more for… those patients that I think are at higher risk’. However, this approach risked inconsistency and omission: ‘In all honesty sometimes I do forget, cause I forget they've got another child’.

### 3.3 Facilitators to Implementing Congenital CMV Prevention Guidelines

Several strategies were suggested (Table 2). At the *system* level, inclusion of CMV in point‐of‐care resources, software templates, and multilingual handouts could support more consistent education. Rebooking patients for follow‐up appointments would also allow more time to cover all the initial antenatal information.

**TABLE 2:** Facilitators to GP implementation of congenital CMV guideline recommendations.

Level 1 subcategory	Level 2 subcategory	Example quotes
Systems level facilitators	CMV resources	‘I love the templates…they help me out a lot’ ‘I usually use the HealthPathways to help me remember things, because there's so many things to remember…I know a lot of GPs use this all for their early pregnancy care’ ‘I like to give handouts… so that they have that visible to them in a resource they can take home and look through and so, if they walk away from consultation going “what was that about again,” (they) have something to refer back to in written form’ Regarding additional supports, or other things that might make it easier to counsel patients about CMV: ‘Yeah, I think that would be the biggest one, giving them some resources to look at themselves’ including ‘different pamphlets in different languages …which is helpful’
	Longer appointments or rebooking patients to follow up	‘I'll ask them to come back and continue the bits that I didn't do, but I like to have 30 minutes’ ‘First antenatal consults are also a lot of information for a patient, so … coming back to things on that second …follow up consult as well’
Practitioner level facilitators	GP self‐created resources or templates	‘giving myself prompts in my… practice software’ ‘I've done a pregnancy book, that I give to patients so they can refer to…they can have a read about it and then flag those questions with me later’
	GP preparation for delivering the education	‘that sounds better, than, you know, just don't kiss your child at all’
Patient level facilitators	Support person present in consultation	‘There's always the utility of having other people in the consult as well who might be a second person to remember what you're saying or discussing whether that's partner or another support person’

At the *practitioner* level, self‐created practice software templates and prepared counselling statements increased confidence. ‘Most of the CMV recommendations (are) now part of my clinic autofill (for) a new pregnancy’, noted one GP. Another reflected: ‘I think it's a nice easy one: “Oh, did you know…?” And then people were happy to know about it’.

At the *patient* level, the presence of a support person during consultations and provision of take‐home resources were seen as improving retention of key messages: ‘There's always the utility of having other people in the consult who might… remember what you're saying, whether that's a partner or another support person’.

## 4 Discussion

GPs surveyed in Australia and internationally have reported that lack of knowledge is a key barrier to sharing CMV hygiene precautions with patients [7, 10]. This study highlights that while an eLearning module improves GP knowledge and confidence, substantial barriers remain to implementing CMV prevention counselling in practice.

Time pressures within standard appointments were considered a major barrier by most of the GPs interviewed, consistent with prior Australian and international research [7, 10]. Insufficient time, coupled with inadequate Medicare reimbursement, limits opportunities to address preventive messages comprehensively. The absence of CMV content in standard GP resources perpetuates inconsistency across providers, undermining the universal approach recommended by national guidelines [5, 6].

GPs were concerned about overwhelming patients with too much information at the initial antenatal visit and identified additional challenges for patients with limited health literacy and/or cultural and linguistic differences. Some GPs also expressed discomfort with the hygiene message, fearing it could provoke anxiety or undermine mother–child bonding. Previous studies of healthcare workers have highlighted similar fears [7, 11].

However, these concerns contrast with evidence demonstrating that most women value receiving CMV advice, prefer to hear it from their general practitioner, and feel empowered rather than distressed by this information [7, 11, 12]. The overlap between perceived patient sensitivities and clinician ambivalence indicates a need for clearer public health messaging and stronger guideline support.

Our findings identified several practical solutions to improving the implementation of CMV prevention education. These include embedding templates and prompts in practice software to act as reminders, preparing short counselling statements, and involving support persons. Staging counselling across multiple visits may help address patient information overload. Take‐home information resources were suggested to improve retention of information, with the availability of multilingual resources and culturally tailored materials critical for equity of care.

Brief digital interventions are effective tools to complement counselling from healthcare professionals [11, 12]. A recent Australian study showed that a two‐minute video significantly improved women's CMV knowledge and willingness to adopt preventive measures [12]. Sharing publicly available information resources and videos, such as those developed by the Cerebral Palsy Alliance [13] could provide a low‐burden, scalable complement to clinician‐delivered information and enhance recall and uptake of key prevention messages.

Policy implications are clear. Updating guidelines in core resources, such as the RACGP Redbook and HealthPathways, to include CMV would provide an authoritative prompt for GPs. Medicare reforms to extend funding for long antenatal consultations could reduce the financial disincentives currently experienced by providers. Broader public health campaigns, leveraging media and community channels, would normalise CMV messaging and complement GP counselling. Integration with digital platforms, including patient apps and clinic portals, could further support equitable access.

### 4.1 Strengths and Limitations

This study provides unique qualitative insights from GPs across multiple states of Australia, providing perspectives on implementing CMV prevention after completing an educational module. However, the small, self‐selected, all‐female sample may not reflect the experiences of the wider GP workforce. Participants also had a professional interest in antenatal care, which may have reduced the diversity of perspectives. Despite these limitations, the findings provide valuable evidence to inform strategies for scaling CMV counselling in general practice.

### 4.2 Future Directions

Future research should explore scalable interventions that integrate seamlessly into busy primary care workflows. Digital innovations, such as automated prompts in practice software and culturally tailored online patient education, hold promise. Public health initiatives targeting childcare centres and antenatal classes could extend the reach of prevention messages beyond the consulting room. Importantly, strategies must be co‐designed with GPs and diverse patient groups to ensure feasibility and accessibility. With universal maternal CMV screening potentially in the pipeline [14], the urgency of embedding CMV counselling into routine practice is growing.

## 5 Conclusion

CMV is a preventable cause of significant childhood disability. While education improves GP knowledge, barriers at systems, practitioner and patient levels continue to limit implementation. Coordinated strategies linking public health campaigns, funding reforms, and integration of CMV content into GP consultation resources are urgently required to support universal CMV counselling in general practice.

## Funding

LH, NR, and TT were supported by the Norman Beischer Medical Research Foundation. KS, EW, and HSS were supported by the Cerebral Palsy Alliance Research Foundation.

## Disclosure

The authors have nothing to report.

## Conflicts of Interest

The authors declare no conflicts of interest.

## Data Availability

The data that support the findings of this study are available from the corresponding author upon reasonable request.
